# Relationship between Maximum Tongue Pressure Value and Age, Occlusal Status, or Body Mass Index among the Community-Dwelling Elderly

**DOI:** 10.3390/medicina56110623

**Published:** 2020-11-19

**Authors:** Hiroki Suzuki, Yasunori Ayukawa, Yoko Ueno, Ikiru Atsuta, Akio Jinnouchi, Kiyoshi Koyano

**Affiliations:** 1 Department of Dentistry, Inouekai Medical Corporation Sasaguri Hospital, Sasaguri Town, Kasuya County, Fukuoka 8112413, Japan; zookey456odpmf@gmail.com (H.S.); youkouiui@yahoo.co.jp (Y.U.); gin741219@icloud.com (A.J.); 2 Section of Implant and Rehabilitative Dentistry, Division of Oral Rehabilitation, Faculty of Dental Science, Kyushu University, 3-1-1 Maidashi, Higashi-ku, Fukuoka 8128582, Japan; koyano@dent.kyushu-u.ac.jp; 3 Division of Advanced Dental Devices and Therapeutics, Faculty of Dental Science, Kyushu University, 3-1-1 Maidashi, Higashi-ku, Fukuoka 8128582, Japan; atyuta@dent.kyushu-u.ac.jp

**Keywords:** tongue pressure, Eichner classification, body mass index, oral frail

## Abstract

*Background and objectives:* In an aging society, the maintenance of the oral function of the elderly is of importance for the delay or prevention of frailty and long-term care. In the present study, we focused on the maximum tongue pressure (MTP) value and analyzed the relationship between MTP and age, occlusal status, or body mass index (BMI). *Materials and Methods:* This one-center observatory study was conducted using a cohort consisting of 205 community-dwelling outpatients over 65 years old. The MTP values of all subjects were measured using a commercially available tongue pressure measurement device and statistically analyzed. In addition, the correlation between MTP value and BMI was analyzed. *Results:* The MTP value decreased with age, especially in subjects classified as Eichner B and C. The difference in occlusal status did not show any statistically significant influence on MTP value. The correlation between BMI and MTP value was indicated in the tested groups other than an age of 65–74 and Eichner A groups. *Conclusions:* Although MTP value decreased with age, the difference in occlusal status did not have an impact on MTP value. The correlation between BMI and MTP value was not shown in the youngest group or a group with sufficient occlusal units. The results presented in the present study may imply that, even if MTP is low, younger age and/or better occlusal status compensate for the inferior MTP value in the cohort studied.

## 1. Introduction

Having a significantly aged population is becoming a global issue and the delay or prevention of their frailty and subsequent demand for long-term care is strongly expected [1]. In Japan, to reduce the demand for long-term care of the elderly, public service programs were introduced in 2006 [2]. Above all, the maintenance of the oral function of the elderly is thought to be important and oral function, such as occlusal force, tongue dexterity, maximum tongue pressure (MTP), masticatory performance, swallowing function, and so on, can be measured under the financial assistance by the insurance system developed by the Japanese government in 2018. MTP has a strong correlation to not just mastication and swallowing [3] but also the amount of skeletal muscles in the whole body [4,5] and mortality [6]. This evidence suggests the importance of the maintenance of tongue pressure for the prevention of systemic frailty. Tongue function has been studied using modalities other than tongue pressure measurement, such as videofluorography and ultrasound diagnostic systems [7,8,9,10,11]. From this viewpoint, a novel tongue pressure measurement device consisting of a disposable oral probe, an infusion tube as a connector, and a recording device was developed [12] and much research has been done using this device, resulting in a lot of useful data [13,14,15]. To date, the device employed in this study is acknowledged as an accredited tongue pressure measurement device from a governmental public insurance system in Japan.

According to a previous report, mean MTP was approximately 32 kPa for people in their 70 s, which is significantly lower than the mean MTP found in younger generations [13]; however, the mean MTP for people in their 80 s has not yet been reported, despite our aging society. In the present study, we measured the average MTP of community-dwelling elderly people, including people in their 80 s.

A previous study reported that tongue pressure appeared during the occlusal phase and reached a peak near the start of opening via measuring tongue pressure against the experimental palatal plate using seven pressure sensors [16]. Since this evidence may imply that the importance of occlusal stability plays a crucial role to generate high tongue pressure, there are no reports regarding this issue as far as the authors know. In the present study, we hypothesized that age and the occlusal units affected the MTP value, and so we studied the relationship between MTP value and age or occlusal status. The relationship between MTP and body mass index (BMI) was also studied.

## 2. Materials and Methods

### 2.1. Participants and Ethical Approval

This study was conducted as a single-centered, retrospective observational study. All participants were 65 years old or older and recruited from the patients who visited the Department of Dentistry, Sasaguri Hospital (Sasaguri Town, Kasuya County, Fukuoka Prefecture, Japan) from June through to November 2018 as outpatients to seek treatment of periodontitis, caries, fabrication/maintenance of dentures, and/or periodical check-ups. In total, 205 participants (76 males and 129 females; mean ± SD age: 78.0 ± 7.6 years) were included in the present study. The patients with dysphagia, who had not received prosthodontic treatment for their edentulous area, and with whom verbal or written communication was difficult, were excluded from this study. The patients with oral soft tissue disease with symptoms suspiciously similar to cancer or other benign or malignant tumor were also excluded.

All participants provided written informed consent to participate in the present study. This study was approved by the ethics committee of Sasaguri Hospital (approval number #19) and was performed in accordance with the ethical standards established in the 1964 Declaration of Helsinki, as revised in 2008.

### 2.2. Examination Measures

The variables analyzed in the present study were sex, age, BMI, the number of remaining teeth, the number of occlusal units at premolar and molar region (occlusal status), and MTP. Ages were divided into three categories, namely 65 to 74, 75 to 84, and 85 to 94 years old. Sex and age were obtained from patients’ medical records. BMI was calculated using the following formula: body weight (kg)/(height (m))^2^. A radiological examination was done for dental or periodontal foci detection, when needed. The data for body weight and height were also obtained from the patient’s medical record. The number of remaining teeth and the number of occlusal units at the premolar and molar regions were checked via visual observation by a dentist (Hiroki Suzuki, H.S.) Occlusal status was divided into three categories, namely A, B, and C, using the Eichner classification [17]. MTP was measured by a dentist (H.S.) using a commercially available tongue pressure measurement device (TPM-02, GC, Tokyo, Japan). The procedure is described in detail elsewhere [18]. In brief, the subjects were asked to compress the balloon, which was connected to a pressure measurement device, by moving their tongue toward the palate for 7 s. The mean value of three measurements was defined as the MTP for each subject. The subjects who were wearing dentures were asked to measure MTP while wearing dentures.

### 2.3. Statistical Analyses

An a priori Shapiro–Wilk test was performed to check for normality. As a result, since normality was not rejected in all cases (*p* > 0.05), parametric tests were employed. In the present study, the following comparisons were performed: (1) the differences of MTP values among 3 age groups; (2) the difference of occlusal status (Eichner classification A, B, or C) within each age group; (3) the differences of MTP values among age groups within each occlusal status. Statistical comparisons were done using one-way analysis of variance (ANOVA) and a post hoc Tukey test for pair-wise multiple comparisons. Pearson correlation coefficient (*r*) was calculated to evaluate the correlations between (1) MTP and BMI within each age group and (2) MTP and BMI within each occlusal status. A value of *p* < 0.05 was considered statistically significant. SPSS for Windows (Ver. 23.0 J, IBM, Armonk, NY, USA) was used for all analyses.

## 3. Results

### 3.1. Participant Characteristics

Study population and average BMI, the number of remaining teeth, and MTP are summarized in Table 1 and Table 2.

### 3.2. The MTP Value in Each Age Group

**Figure 1:**
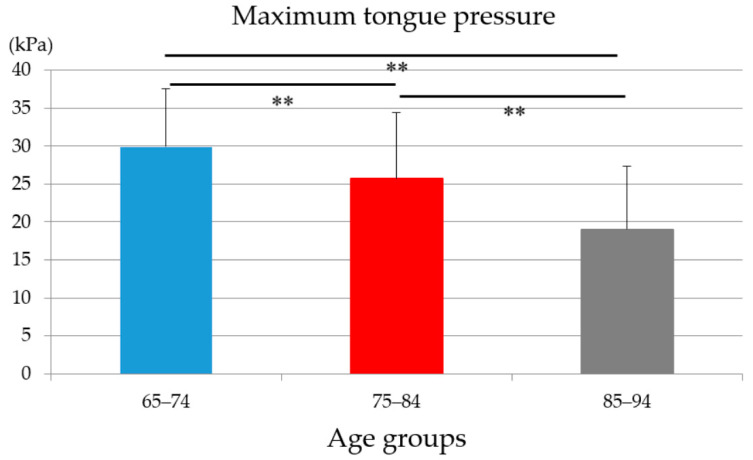
The maximum tongue pressure values of three age groups (ANOVA, ** *p* < 0.01).

MTP values were 29.88 ± 7.67 in ages 65–74, 25.72 ± 8.69 in ages 75–84, and 19.03 ± 8.31 in ages 85–94. There were statistically significant differences among three age groups (ages 65–74 vs. ages 75–84: *p* = 0.005; ages 65–74 vs. ages 85–94; ages 75–84 vs. ages 85–94: *p* < 0.001) (Figure 1).

### 3.3. The Difference of MTP Value among Age Groups within Each Occlusal Status (Eichner Classification)

**Figure 2:**
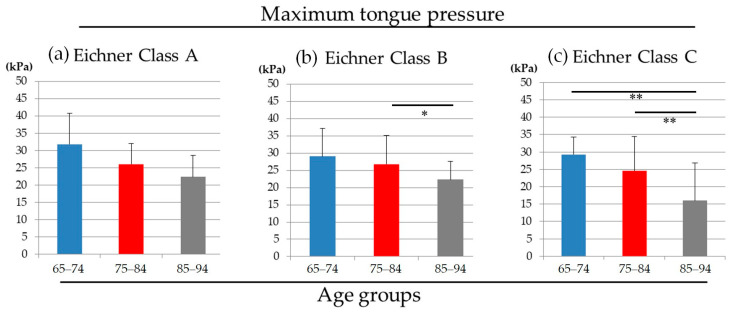
The maximum tongue pressure values of three age groups in (**a**) Eichner class A, (**b**) class B and (**c**) class C. (ANOVA, * *p* < 0.05, ** *p* < 0.01).

In Eichner classification class A, no significant differences were reported among three age groups. In class B, there was a statistically significant difference between ages 75–84 and ages 85–94 (*p* = 0.025). In class C, there were statistically significant differences among the three age groups (*p* < 0.001) (Figure 2).

### 3.4. The Difference of MTP Value among Occlusal Status within Each Age Group

**Figure 3:**
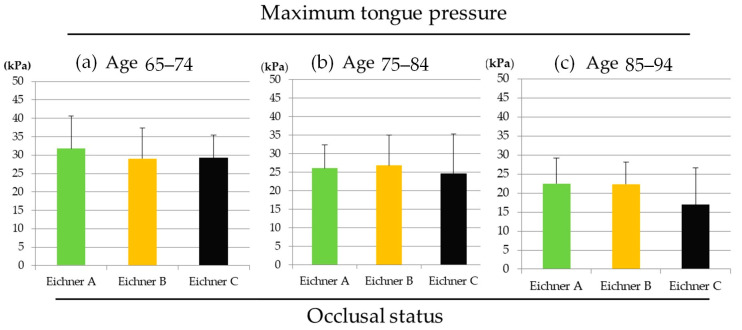
The maximum tongue pressure values of three occlusal status in (**a**) ages 65–74, (**b**) ages 75–84 and (**c**) ages 85–94 groups. (ANOVA, *p* > 0.05).

In the ages 65–74 group, there were no statistically significant differences in MTP values among Eichner classes A, B, and C. The same tendencies were observed in the other two age groups (Figure 3).

### 3.5. Correlation Coefficients between MTP and BMI within Each Age Group and MTP and BMI within Each Occlusal Status

**Figure 4:**
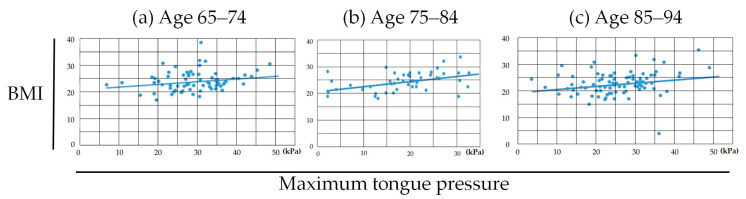
Pearson correlation coefficient between MTP and BMI in (**a**) ages 65–74, (**b**) ages 75–84 and (**c**) ages 85–94 groups. Statistically significant correlations were found in the ages 75–84 group (**b**) (*r* = 0.221, *p* = 0.041) and the ages 85–94 group (**c**) (*r* = 0.440, *p* = 0.002).

Between MTP value and BMI within each age group, statistically significant positive correlations were found in the ages 75–84 group (*r* = 0.221: weak correlation, *p* = 0.041) and the ages 85–94 group (*r* = 0.440: moderate correlation, *p* = 0.002) (Figure 4).

**Figure 5:**
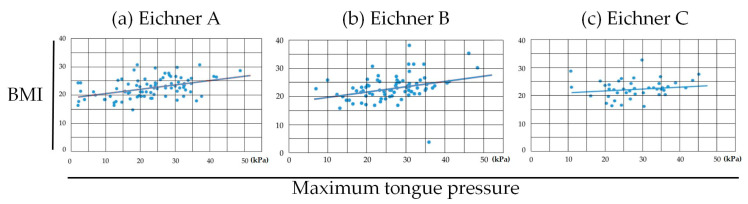
Pearson correlation coefficient between MTP and BMI in (**a**) Eichner class A, (**b**) class B and (**c**) class C. Statistically significant correlations were found in Eichner class B group (**b**) (*r* = 0.330, *p* = 0.003) and Eichner class C group (**c**) (*r* = 0.438, *p* < 0.001).

Between MTP and BMI within each occlusal status, statistically significant positive correlations were found in Eichner class B (*r* = 0.330: weak correlation, *p* = 0.003) and Eichner class C (*r* = 0.438: moderate correlation, *p* < 0.001) (Figure 5).

## 4. Discussion

In the present study, a commercially available tongue pressure measurement device was used to measure MTP value. The device used here was composed of a disposable oral probe, an infusion tube as a connector, and a recording device. There are some other kinds of tongue pressure devices, such as the Iowa Oral Performance Instrument [19] and the KayPENTAX Digital Swallowing Workstation tongue pressure measurement device [20]. The device employed in the present study was considered to be equivalent to these two devices [21]. The validity of this device was well-studied by a previous study and this device was concluded to be a simple screening tool for frailty because MTP performance was independently associated with frailty [22].

The mean MTP of the cohort analyzed in the present study was 25.67 kPa. This value was approximately 20% lower than that of the people in their 70 s (31.9 kPa), as reported previously [13]. In the present study, the mean MTP value of the ages 65–74 group was 29.88 kPa, which was a similar value to the previous study, but the mean MTP value was 25.72 kPa in the ages 75–84 group and 19.03 kPa in the ages 85–94 group. In addition, the MTP value decreased with age. This suggests that aging has a strong impact on decreasing the MTP value. In addition, an MTP value less than 30 kPa was diagnosed to be oral frailty in Japan, irrespective of age, without any strong evidence to support this cut-off value. Our results suggest the importance of reconsidering this cut-off value corresponding to patients’ age.

The decreasing tendency of the MTP value in an age-dependent manner was enhanced in Eichner B and C groups. In the present study, all subjects were asked to wear their removable prostheses while measuring MTP if they usually used it. We did not separate fixed and removable prosthesis while classifying Eichner classification; thus, further investigation is required, though Eichner class B and C subjects were supposed to indicate an increased chance of a given person wearing a removable prosthesis, which may be attributable to inferior occlusal force. A report indicated that removable denture use was related to decreased masticatory performance [23]. Another study reported that MTP was significantly associated with maximum occlusal force and tooth loss [24]. These reports reinforced the hypothesis that lower occlusal force ascribed from the loss of occlusal units (Eichner B and C) and the subsequent wearing of a removable denture reduced the MTP value.

There were no statistically significant differences in MTP values among three occlusal statuses within each age group. In conjunction with the deteriorating tendency of MTP value with age (Figure 1 and Figure 2), aging were supposed to have more impact on MTP value than occlusal status; however, to draw this conclusion, further analyses are mandatory.

Regarding the correlation between BMI and MTP, statistically significant correlations were observed in ages 75–84, ages 85–94, and Eichner B and C groups. This result indicates that the relatively younger generation and relatively better occlusal status within this cohort could maintain their BMI regardless of their MTP value. From these results, it can be speculated that people who are younger or have a better occlusal status can compensate for the weakness of the lower MTP value. If this speculation is true, the maintenance of occlusal status seems to be important for systemic health because aging and decrease of MTP value are difficult to avoid.

According to a previous report, the number of functional teeth, but not the number of present teeth, was a significant independent mortality risk factor [25]. In this study, functional teeth included the functioning present teeth plus the number of artificial teeth on removable dentures being worn during the oral examination, dummies on fixed partial dentures, and implant-supported artificial teeth. This denotes that either artificial or natural teeth, with occlusal function, play a crucial role in health. This may agree with our results, as indicated in Figure 3, that the difference of occlusal status has less impact on MTP value partly because all subjects in the present study wore removable dentures.

The limitation of the present study is that our cohort included only outpatients. This means that this cohort consisted of relatively healthy subjects, regardless of their age. The lack of general health data and drug intake in the participants in the present study should also be taken into account because these variables vary among the elderly and may influence MTP values. In addition, in the present analysis, we did not check the periodontal status. This may have an impact on occlusal status or MTP. Additionally, as described above, we did not separate fixed and removable partial dentures. This may affect the result. Studies with a larger cohort and an intent to measure the MTP value, separating removable and fixed partial denture wearers, are expected.

Another limitation includes the lack of consideration of socioeconomic status. Since a previous report indicated that more affluent people tended to receive dental treatment [26], low socioeconomic status may affect the occlusal status and MTP. On the contrary, in Japan, general dental treatment, including fabricating a removable denture, is covered by the national health insurance system, and patients easily and inexpensively undergo medical/dental treatment. Thus, our conclusions should be used with caution when it is extrapolated to other countries, especially those with other health insurance systems.

## 5. Conclusions

Within the limitation of the present study, we concluded that MTP value decreased with age, especially in subjects classified Eichner B and C. The difference in occlusal status did not show any statistically significant influence on MTP value. The correlation between BMI and MTP value was indicated in the tested groups other than ages 65–74 and Eichner A groups. This may imply that, even if MTP is low, younger age and/or better occlusal status compensate for the inferior MTP value in the cohort studied in the present study.

## Figures and Tables

**Table 1:** The number of subjects in each age group (left) and Eichner classification (right).

Age (Years Old)	*n*	Eichner Classification	*n*
65–74	74	A	42
75–84	84	B	78
85–94	47	C	85
Total	205	Total	205

**Table 2:** The averages of body mass index (BMI), the number of remaining teeth, and maximum tongue pressure (MTP) in all subjects.

	Average ± SD
Age	78.00 ± 7.64
BMI	22.42 ± 4.00
The number of remaining teeth	16.11 ± 9.31
MTP (kPa)	25.67 ± 9.16
